# A Multimodal Transformer Model for Recognition of Images from Complex Laparoscopic Surgical Videos

**DOI:** 10.3390/diagnostics14070681

**Published:** 2024-03-23

**Authors:** Rahib H. Abiyev, Mohamad Ziad Altabel, Manal Darwish, Abdulkader Helwan

**Affiliations:** 1 Applied Artificial Intelligence Research Centre, Department of Computer Engineering, Near East University, 99132 North Cyprus, Turkey; mohamadziad.altobel@neu.edu.tr (M.Z.A.); manal.khaled.darwish@gmail.com (M.D.); 2 Department of Health, Medicine and Caring Sciences, Linköping University, 581 85 Linköping, Sweden; abedelkader.helwan@liu.se

**Keywords:** transformer, laparoscopic videos, ViT, BERT, transformer encoders, text and image embedding

## Abstract

The determination of the potential role and advantages of artificial intelligence-based models in the field of surgery remains uncertain. This research marks an initial stride towards creating a multimodal model, inspired by the Video-Audio-Text Transformer, that aims to reduce negative occurrences and enhance patient safety. The model employs text and image embedding state-of-the-art models (ViT and BERT) to assess their efficacy in extracting the hidden and distinct features from the surgery video frames. These features are then used as inputs for convolution-free Transformer architectures to extract comprehensive multidimensional representations. A joint space is then used to combine the text and image features extracted from both Transformer encoders. This joint space ensures that the relationships between the different modalities are preserved during the combination process. The entire model was trained and tested on laparoscopic cholecystectomy (LC) videos encompassing various levels of complexity. Experimentally, a mean accuracy of 91.0%, a precision of 81%, and a recall of 83% were reached by the model when tested on 30 videos out of 80 from the Cholec80 dataset.

## 1. Introduction

Advancements in technology have led to the improvement of computer-assisted interventions (CAIs) in surgical procedures [1]. However, the increasing complexity of the operative environment has made it necessary to process and integrate data flows from various technologies to enrich surgical practice. This can help support surgeons in making decisions, anticipating possible complications, and enhancing cooperation between multidisciplinary OR teams. Additionally, surgical workflow recognition can benefit OR resource management optimization, automatic report generation, surgeon training, and operative skill assessment [2].

The recognition of surgical activities and the conceptualization of surgical workflow are heavily dependent on accurate surgical tool detection [1,3]. Various techniques and methodologies have been employed to identify surgical tools [4,5]. Early approaches used radio-frequency identification (RFID) systems to acquire tool use signals [5]. However, this technique requires the installation of specific sensors and instruments that may interfere with the intervention workflow. As a result, modern alternatives such as image-based laparoscopic video signal approaches have been investigated. Visual features in different color spaces can be employed to separate tool pixels and identify tool types [6]. Other studies have used features of ORB (Oriented FAST and Rotated BRIEF), SIFT (Scale Invariant Feature Transform), and SURF (Speeded Up Robust Features) to classify surgical tools [7,8]. A detailed discussion of the related works [6,7,8,9,10,11,12,13,14] is shown in the next section.

In this work, we developed a Transformer based framework inspired by VATT [15] for the laparoscopic surgery videos phase recognition. This framework model incorporates state-of-art methods such as Vision Transformer (ViT) [16] and Bidirectional Encoder Representations from Transformers (BERT) [17] for the extraction of image and text embeddings from surgical videos and their text descriptions, respectively. The core of this framework is that it uses two Transformer encoders that are trained separately using different input modalities in order to extract modality-specific representations or feature vectors of every input modality. This allows the model to learn the specific features of each modality without being biased by the other modality which can consequently lead to better performance on downstream tasks that require understanding both modalities. Once these Transformer encoders are trained, they are combined to form a joint multimodal representation. This is achieved by concatenating the hidden states of the two encoders and using them as inputs for a separate multilayer perceptron (MLP) which learns to recognize the surgery phase using stochastic gradient descent [18].

The contributions of this paper include: (1) Evaluation of one of the state-of-art multimodal text and image models for laparoscopic surgical phase classification. (2) Robustness assessment of text and image embedding models for surgical phase classification in multiple types of laparoscopic procedures. (3) Evaluation of ViT and BERT models for acting as text and image embedding extracting models. (4) Evaluation of the effect of employing the multimodal projection head as a joint embedding space for combining the image and text hidden states learned by Transformer encoders. (5) Evaluation of the impact of using two separate Transformer encoders for every embedding vector instead of using only one. (6) Systematic evaluation of the whole model on a dataset of laparoscopic videos of seven different phases.

The paper is organized as follows. Section 2 includes the description of VATT and its training mechanism. Section 3 presents the architecture of the proposed VATT and its learning. The dataset used in the paper is described. Section 4 gives implementation details of the model and obtained important results. Section 5 gives the conclusions of the paper.

## 2. Related Works

The expansion of deep learning approaches in object classification tasks has directed medical researchers to explore convolutional neural networks (CNNs) [6,9]. However, the paucity of labeled datasets has hindered wider exploration of the potential of CNNs for analyzing laparoscopic images. The Cholec80 dataset [3], which contains labeled laparoscopic videos of 80 surgeries, was made available to researchers in 2017 [3]. The first utilization of Cholec80 was carried out by training a CNN model, EndoNet [3], to learn visual features for recognizing surgical tools and phases. Subsequent studies alleviated the imbalanced dataset problem by applying loss weights and resampling strategies. In addition to spatial information captured by the CNN, other studies have leveraged temporal dependencies along the video sequence using long short-term memory (LSTM) [10], convolutional LSTM [11], gated recurrent unit (GRU) [12], or graph convolutional networks (GCNs) [13].

The proposed methods [4,5,6,7,8] in previous studies show good performance for detecting and classifying surgical tools. However, the recognition phase of the surgery being carried out is still considered a challenging task [6]. The surgery videos can be affected by many factors which can cause distortion to the model such as tools being used during surgery, noise, and smoke in the case of some surgeries, but more importantly the similarities between the phases of one specific surgery. As a result, the robustness of automatic surgery phase recognition in different datasets has not yet been reached.

Several studies on laparoscopic videos have addressed the generalization capability of deep learning models [6]. Bar et al. [14] studied the generalization of a deep model consisting of CNN-LSTM for surgical phase recognition. A reduction of about 10% in accuracy was reported on videos from an unseen hospital. Table 1 summarizes some of the discussed literature focusing on surgical phase recognition and tool detection using conventional and machine learning methods.

## 3. Materials and Methods

### 3.1. Video-Audio-Text Transformer (VATT)

VATT, which stands for Video-Audio-Text Transformer, is a cutting-edge framework designed to acquire multimodal representations from unlabeled data [15]. What sets VATT apart is its utilization of convolution-free Transformer architectures to extract comprehensive multidimensional representations. These representations possess a wealth of information that can be advantageous for various downstream tasks. The remarkable aspect of VATT is that it can take raw signals, such as video, audio, and text, as inputs and generate representations that are applicable to a wide range of tasks, eliminating the need for task-specific training. To accommodate the differences among modalities, VATT establishes a shared space, while employing noisy contrastive estimation to train the model effectively [16].

VATT’s architecture is built upon two well-known models, BERT and ViT [15]. However, a notable distinction in the VATT architecture is the separate tokenization and linear projection layers for each modality. This modification aligns with ViT’s approach of making minimal changes to facilitate weight transfer across frameworks and tasks.

Through this architecture, VATT is capable of generating joint representations that effectively capture the interactions between input signals from different modalities. Consequently, VATT can produce a unified representation from diverse modalities, enhancing its efficiency in processing complex inputs. Moreover, the multidimensional representations generated by VATT’s architecture possess sufficient richness to benefit a range of downstream tasks, including speech recognition, image captioning, and video retrieval [15].

### 3.2. Training the VATT

VATT [15] is trained with noise contrastive estimation (NCE) [19] which is a statistical method employed in the field of machine learning to approximate the parameters of intricate probability models. This training method proves particularly advantageous for models where the calculation of the normalization constant, also referred to as the partition function, is arduous or computationally demanding. By utilizing NCE, the task of parameter estimation for a complex probability model is transformed into a binary classification problem. The underlying concept involves training a binary classifier to differentiate between genuine data and noise samples generated from the model. Subsequently, the parameters of the model are estimated by maximizing the likelihood of the real data under the model. NCE has demonstrated its computational efficiency and effectiveness in various domains, including natural language processing and computer vision.

NCE turns the problem of estimating the parameters of a complex probability model into a binary classification problem. The idea is to train a binary classifier to distinguish between the true data and noise samples generated from the model. The parameters of the model are then estimated by maximizing the likelihood of the true data under the model [15,19]. The NCE loss function is defined as:

(1) $$L_{NCE}=-\frac{1}{n}\sum_{i=1}^{n}log\frac{p(x_{i})}{p\left(x_{i}\right)+k\cdot \;p_{n}(x_{i})}$$

where $p_{n}(x_{i})$ is the noise distribution, and *k* is the number of noise samples per true sample. The NCE method has been shown to be effective for training models with a large number of classes, such as the VATT model.

### 3.3. The Proposed Model

**Figure 1:**
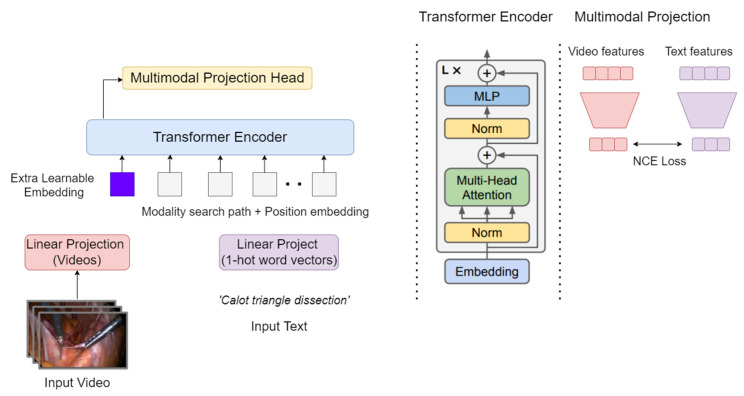
The proposed model architecture and supervised multimodal learning strategy involve linearly projecting each modality into a feature vector and feeding it into a Transformer encoder. A semantically hierarchical common space is defined to account for the granularity of different modalities, and the contrastive loss method is employed to train the model. The extra learnable embeddings refer to additional vector representations introduced in the model architecture. These embeddings capture specific information that goes beyond the base embeddings used for video, audio, and text modalities.

In this paper, we employed similar VATT architecture to build a model that can recognize the surgical phases from a video. The proposed model (Figure 1) takes raw signals as inputs from the laparoscopic videos and extracts multimodal representations that are rich enough to benefit a variety of downstream tasks. The developed model uses a joint embedding space to represent the input signals, which allows the model to learn the relationships between different text and image modalities. The joint embedding space is created by combining the image and text hidden states or feature vectors, learned by Transformer encoders, using a multimodal projection head [15,20].

First, the image embeddings are extracted from the raw video laparoscopic frames using a convolution-free vision Transformer (ViT), while the text embeddings are extracted from the raw text using a language Transformer (BERT). The Vision Transformer [16] is based on the Transformer architecture [21], which is a type of neural network that is particularly well-suited for processing sequential data. A pre-trained version of the BERT model [17] was used directly to extract the text embeddings. On the other hand, for image embedding extraction, a pre-trained version of ViT was fine-tuned. The Transformer encoders were then trained separately using a multimodal contrastive loss function [22], which pushes the embeddings of similar modalities to be close together in the joint embedding space, while pushing the embeddings of dissimilar modalities apart. The multimodal contrastive loss function is defined as:

(2) $$L_{MCL}=-\frac{1}{n}\sum_{i=1}^{n}log\frac{exp(sim\left(x_{i},y_{i}\;\right)/\tau }{\sum_{j=1}^{n}exp(sim\left(x_{i},y_{i}\;\right)/\tau }$$

where $x_{i}$ and $y_{i}$ are the embeddings of the *i*-th image and text pair, respectively, $sim\left(x_{i},y_{i}\right)$ is the cosine similarity between the embeddings, and *τ* is a temperature parameter that controls the sharpness of the distribution.

**Figure 2:**
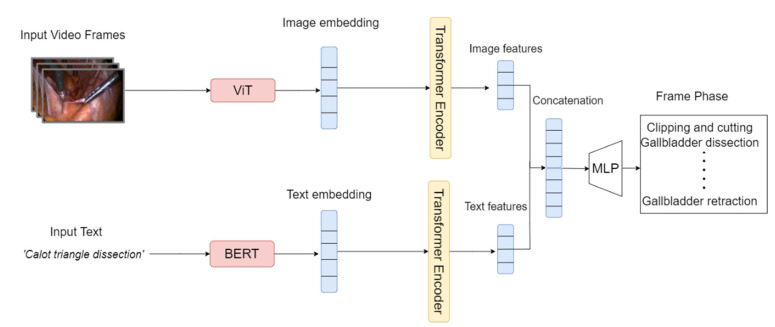
Extracting the text and image embeddings using BERT and ViT, respectively.

Figure 2 shows how the model employs two separate Transformer encoders, one for each modality: image and text. As seen, each encoder processes its respective input data independently and extracts modality-specific representations. These representations are then combined across modalities using a semantically hierarchical common space to form a unified representation that captures the relationships between the different modalities. A breakdown of how the model architecture handles each modality is as follows:

Video: The video encoder (ViT) takes as input raw video frames and extracts a sequence of feature vectors from each frame. These feature vectors are then fed into the Transformer encoder, which employs self-attention and positional encoding to learn a hierarchical representation of the video sequence.

Text: The text encoder (BERT) takes as input a sequence of words or characters and represents the text as a sequence of token embeddings. These token embeddings are then fed into the second Transformer encoder, which learns a representation of the text that captures the semantic and syntactic relationships between words.

## 4. Experimental Setup

### 4.1. Implementation Details

Our method is implemented with the TensorFlow framework. All experiments were carried out on a GeForce GTX 1640Ti graphical processing unit (GPU). In contrast to the AVTT model [15], our temporally rich spatial feature extractor consists only of two transformer embedding models. The frame (image) encoder uses the ViT-B/16 architecture, which is pretrained on ImageNet1K (IN1k) [23] with an input image size of 248 × 248 pixels and output size of Ds = 768D representations. The second Transformer encoder is a pre-trained BERT model. The ViT feature extractor was trained with Stochastic Gradient Descent for 35 epochs, with a 5-epoch warmup [24] and cosine annealed decay. After training, the text and image embeddings were fed to the Transformer encoders which were separately trained for 50 epochs with SGD and weight decay of 1 × 10^−5^, learning rate of 0.001, and a 5-epoch warmup. During training, the model was fed with 50 video frames for training while the rest were kept for testing. Both transformer encoders have a 12-layer structure. The learning parameter optimization was based on their impact on the model performance on a held-out validation set. Different configurations were compared and the one that achieved the best performance on the chosen evaluation metric was reported.

The multimodal projection head serves as the final layer of each Transformer encoder. Its purpose is to connect the modality-specific representations learned by the encoders with the unified representation used for downstream tasks. To achieve this, the multimodal projection head applies linear projections to the modality-specific representations and combines them across modalities in a semantically hierarchical common space. This hierarchical common space ensures that the relationships between the different modalities are preserved during the combination process. In the training process of Transformer encoders, the contrastive loss plays a crucial role. This optimization technique enables each model to learn meaningful representations from the input data. It computes the loss for each pair of examples (images and texts), encouraging similar pairs to have a smaller distance and dissimilar pairs to have a larger distance. By distinguishing between pairs of text and image embeddings, the model can effectively capture the relationships between the different modalities.

The multimodal projection head structure that was used in the work is the same one used in VATT [15], which is practically a two-stage process. 1. Feed-forward network: The joint representation of the text and image embeddings from the Transformer encoders is first passed through a feed-forward network. This network consists of two fully connected layers with a ReLU activation function between them. The output of the feed-forward network is a new representation of the data that is more compact and easier to process. 2. Linear layer: The output of the feed-forward network is then passed through a linear layer. This layer converts the representation into a vector of desired size, which is then used as input for the MLP.

### 4.2. Cholec80 Dataset

**Figure 3:**
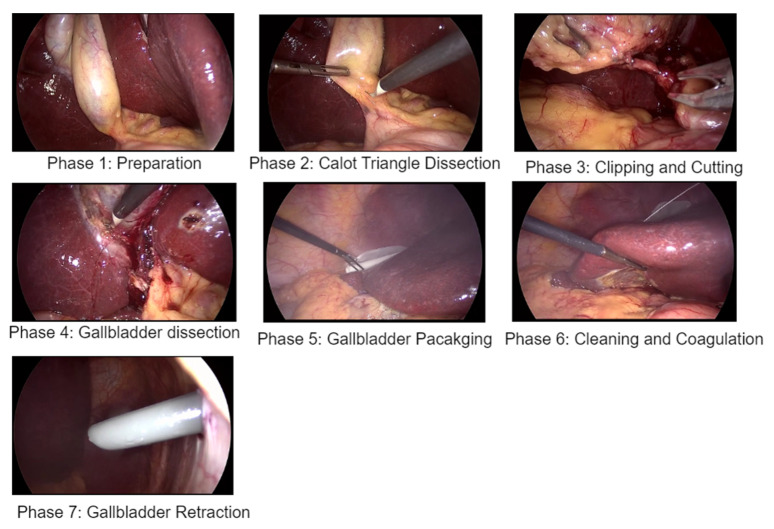
Samples of surgery video frames of the seven different phases from Cholec80 dataset.

**Figure 4:**
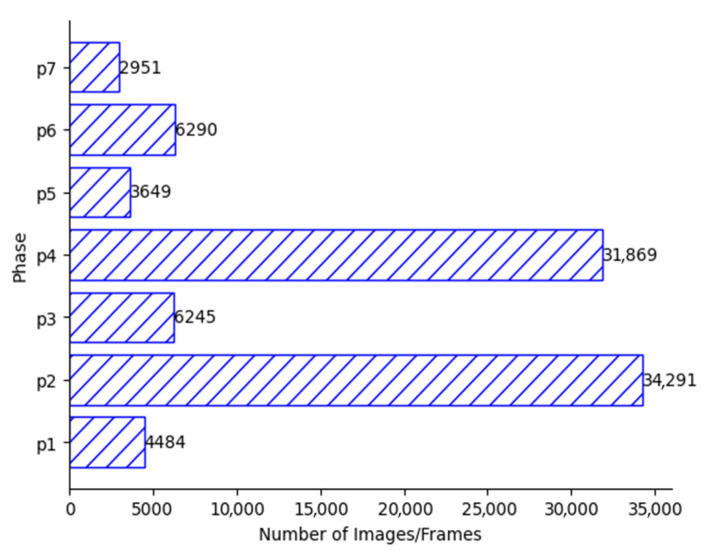
Number of images/frames for each phase of Cholec80 dataset.

In 2017, a dataset comprising 80 videos of cholecystectomy procedures conducted by thirteen surgeons was made publicly accessible [3]. These videos were recorded at the University Hospital of Strasbourg in France, with a data acquisition rate of 25 Hz. Among the videos, three had a resolution of 1920 × 1080, while the remaining videos had a resolution of 854 × 480. The median duration of the videos was found to be 34.9 min, with a minimum duration of 12.3 min and a maximum duration of 99.9 min. The dataset underwent manual labeling for surgical tools at a rate of 1 Hz and surgical phases at a rate of 25 Hz (25 frames per second). The frames are provided with manual annotations carried out by surgeons indicating the surgical phase or activity of each video frame and the name of tools appearing in the scene. Cholec80 videos consist of seven phases: preparation, Calot’s triangle dissection, clipping and cutting, gallbladder dissection, gallbladder packaging, cleaning and coagulation, and gallbladder retraction. For this work, we only use phase annotations, as our model task is to recognize the surgical video phase at every frame. Figure 3 shows a sample of the seven different phases in the Cholec80 dataset. These frames were all taken from the same videos that include frames of all different phases. Table 2 shows the number of videos used in training and testing of the model. Table 3 shows the surgery phases and their corresponding durations. Figure 4 shows the number of images/frames for each surgery phase in the Cholec80 dataset [3].

## 5. Results and Discussion

### 5.1. The Text and Image Embedding Extraction Models

**Figure 5:**
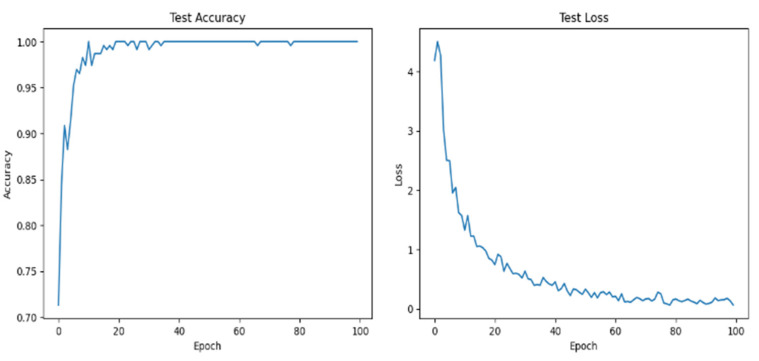
Accuracy and loss variations of ViT trained to extract image embeddings.

In Figure 5, the ViT training results are shown. As mentioned earlier, a pre-trained version of ViT was finetuned using the entire Cholec80 dataset to extract embeddings from images/frames. The output layer of the pretrained ViT was removed to match our embedding layer output. In ViT, the final layer before the output is often a global average pooling layer that aggregates spatial information across the entire image. This operation produces a fixed-size feature vector, which is then usually followed by one or more fully connected layers leading to the output layer. Once trained, text embeddings were extracted using BERT, which was employed directly without finetuning.

### 5.2. The Transformer Encoders

After the extraction of text and image embeddings, both passed through different Transformer encoders which were trained using contrastive loss to extract text and input features or so-called hidden states. These extracted features were then passed through the multimodality projection head and then final combined hidden states were fed into the MLP, which was trained to classify the phase of the surgery. To ensure generalizability, we employed k-fold cross validation (CV) with k = 5 for robust evaluation. The evaluation metrics represent the average values of all.

**Figure 6:**
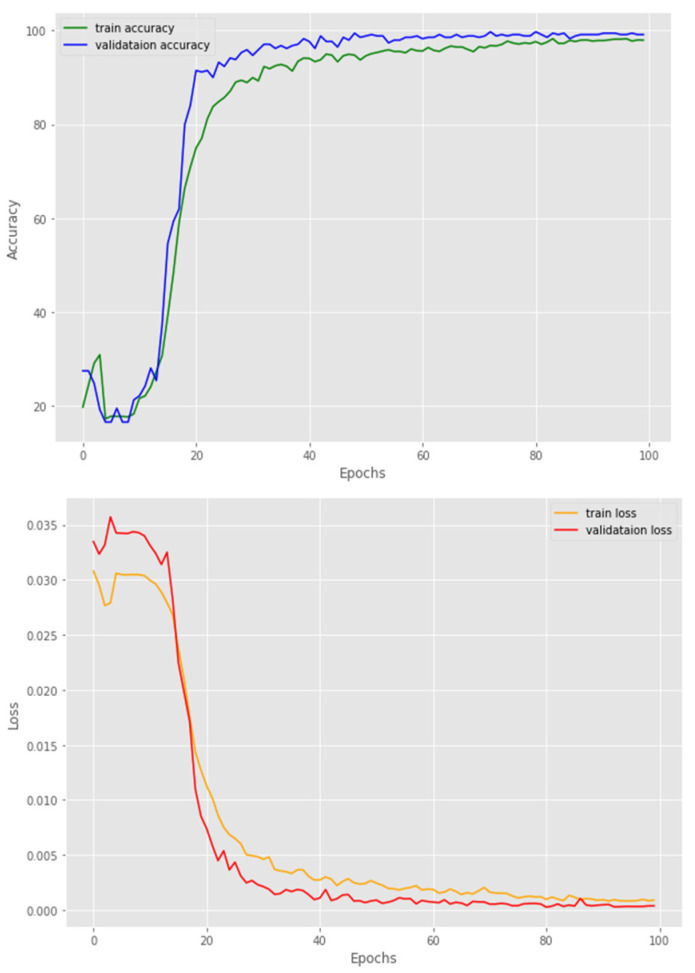
Accuracy and loss variations of the MLP trained to classify surgery phases.

Figure 6 shows the testing results of the MLP trained with extracted text and image embeddings as input data to classify the surgery phases. As seen in Figure 5, the MLP reached a high accuracy rate of 91% during testing/validation. The performance of the automatic recognition of surgical phases is shown in Table 4. In this table, the weighted average precision and recall were respectively 0.81 and 0.83 for the surgical phase recognition. The average results in Table 4 represent the results (accuracy, precision, and recall) achieved across all folds. The standard deviation of every metric across all folds is also shown in this Table 4.

**Figure 7:**
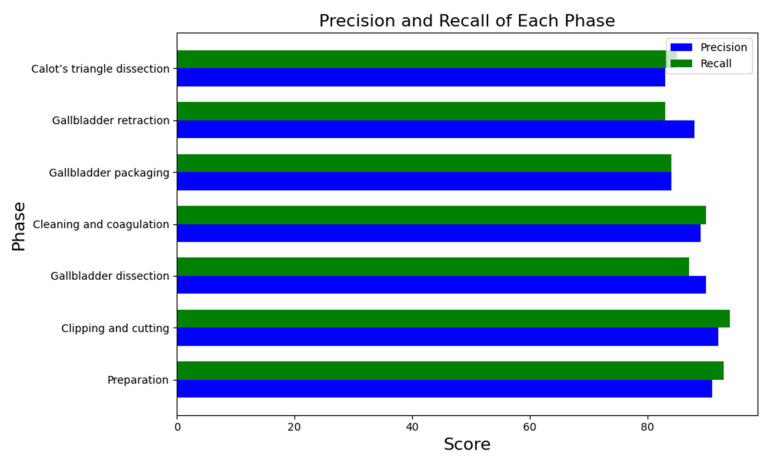
Precision of every phase.

Figure 7 shows the testing precision and recall achieved by the model for every surgery phase.

### 5.3. Comparison

The model developed in this work uses two Transformer encoders for text and image embedding. In order to showcase the importance of using two separate Transformer encoders for text and image embedding, we built a model similar to the latter but with only one Transformer encoder. In here, text and image embeddings are extracted and concatenated and then fed into a Transformer encoder which outputs one feature vector. This vector is then used to train the MLP. We trained the whole model using the same setting used to train our original model; however, this one-Transformer encoder model was trained using cross entropy loss, as contrastive loss is not needed in this case. The results are shown in Table 5. As expected, the performance of the model decreased when one Transformer encoder was used.

For more interpretability of the model performance, our results are compared with other state-of-art models trained and tested on the Cholec80 dataset. The results of this comparison are shown in Table 6.

### 5.4. Inference

**Figure 8:**
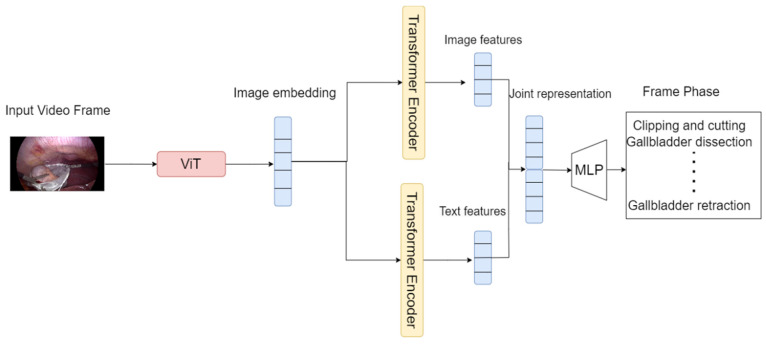
The inference model structure after being trained.

The developed surgery phase recognition framework comprises several Transformer based models which makes testing it on a video or a frame seem complicated. However, testing on a frame is only one forward run process through the whole model, similar to testing on 30 videos. The steps to use the inference model are shown in Figure 8 and they are as follows: A video is divided into frames. A frame is fed into both ViT where image embeddings are extracted. These extracted embeddings are then fed into different Transformer encoders which outputs two feature vectors. These vectors pass through a multimodality projection head and hidden states from every encoder and are then produced and combined. The combined feature vector is finally passed into the MLP which classifies its phase using Softmax.

### 5.5. Discussion

The main goal of this study was to present an artificial intelligence-based multimodal model that can assist medical surgeons in improving their surgery outcomes by acting as a “second set of eyes”. The aim of this research was to develop an artificial intelligence (AI)-based multimodal model that could automate the process of phase recognition in laparoscopic cholecystectomy. The presented model demonstrated a high level of accuracy, successfully identifying surgical phases with an overall accuracy rate of 91.0%. This level of accuracy is comparable to several works in the literature (Table 3). Notably, our model was able to detect laparoscopic surgical phases even in procedures with complications such as major bleeding, major bile leakage, major duct injury, and gallbladder perforation.

We also opted to use a paired *t*-test as a suitable statistical analysis tool to compare these two Transformer models with different encoder numbers. This statistical analysis is shown in Table 7. The level of significance (alpha) was set to 0.05 as we sought to confirm that both models are statistically different to each other. The *p*-value indicates the probability of observing such a difference by chance. A low *p*-value (typically less than 0.05) suggests a statistically significant difference between the two models’ performance.

As a result, if the obtained *p*-value is less than the alpha value then the null hypothesis can be neglected. Table 7 also shows the t-statistic (6.12) which represents the standardized difference between the means of the paired data (differences in performance metrics between the two Transformer models). As seen, the paired *t*-test revealed a statistically significant difference between the one-encoder and two-encoder models.

**Figure 9:**
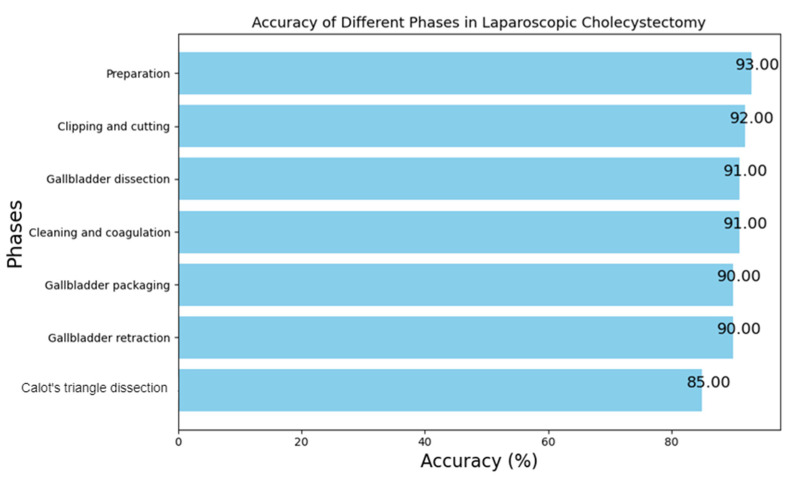
Accuracies achieved by the model for every surgery phase.

Different surgical phases carry different levels of significance. For instance, the successful recognition of the Calot’s triangle dissection, the clipping and cutting phase, and the gallbladder dissection are paramount for ensuring the safety of the patient. Conversely, misidentifying the Calot’s extraction and preparation phase has a relatively lower impact on patient safety. Figure 9 shows the accuracies achieved by the model at every phase. It is worth noting that our multimodal model has achieved an impressive accuracy rate of 92% in the clipping and cutting phase, and 90% in the gallbladder retraction thereby providing strong support for the critical surgical phases. It is also important to mention that the model achieved the highest accuracy in the preparation phase, which is due to the simplicity of the frames in this phase in which no apparatus or tools are found which makes it easy for the model to recognize.

Additionally, the developed model demonstrated consistent high performance even in the face of unfavorable circumstances, showcasing its overall resilience during laparoscopic surgery procedures. Surprisingly, these variations did not hinder the model’s ability to accurately recognize different phases, achieving an impressive overall accuracy range of 85–93%. These outcomes highlight the system’s adaptability and reliability.

The strengths of such a multimodal model include its ability to learn complex relationships such as temporal dependencies, which is crucial in this application, as surgical phases often have a specific order. Hence, the model can capture these temporal relationships by analyzing the sequential nature of the data within each modality. Moreover, such a Transformer-based model can excel at capturing long-range dependencies across different modalities in which extracting subtle cues from earlier stages (e.g., preparation) might help it predict later phases (e.g., clipping and cutting).

## 6. Conclusions and Future Works

The multimodal model presented in this study showed remarkable performance regarding the recognition of laparoscopic surgical videos. However, several limitations are associated with this study. The employed multimodal model used in this research was specifically trained to identify normal surgical phases, such as preparation and Calot’s triangle dissection, in videos. However, the presence of adverse events in these videos may have had an impact on the model’s performance. During adverse events, the scenes captured may not be directly related to the ongoing surgical phase, which could have affected the accuracy of the AI model. To address this limitation, future work should focus on adapting the AI model to also recognize adverse events, which could potentially enhance its performance. Furthermore, it is important to note that some adverse events were rare occurrences. To improve the model’s ability to correctly identify such events, it would be beneficial to have additional examples of these rarer adverse events for training and evaluating purposes. Another limitation of this study is the non-real-time nature of the system. As a result, it cannot provide safety indications during the actual surgical procedure. These limitations highlight the need for further research and development in order to enhance the effectiveness of AI-assisted surgical recognition.

## Figures and Tables

**Table 1:** General information summary of all related works.

Author	Method	Task	Dataset
Twinanda et al. [3]	CNN	Surgical phase recognition	Cholec80 [3]
Miyawaki et al. [5]	RFID technology	Surgical tool recognition	-
Alshirbaji et al. [6]	CNN (DenseNet-121)	Surgical tool recognition	Cholec80 [3], EndovisChole [6], and Gyna08 [6]
Doignon et al. [7]	Discriminant color feature with respect to intensity variations and specularities.	Surgical tool segmentation	-
Primus et al. [8]	Object detection, SVM classifiers and ORB features	Temporal segmentation of surgical phases	Cholec80 [3]
Mishra et al. [10]	CNN and Long Short-Term Memory network (LSTM)	Identifying surgical tools	Cholec80 [3]
Nwoye et al. [11]	CNN + Convolutional LSTM (ConvLSTM)	Surgical tool tracking	Cholec80 [3]
Namazi et al. [12]	Recurrent Convolutional Neural Network (RCNN)	Surgical tool recognition	Cholec80 [3]

**Table 2:** Cholec80 dataset and training/testing scheme.

Cholec80 Dataset	
Number of videos	80
Training	50
Testing	30

**Table 3:** List of phases in the Cholec80 including the mean ± std of the duration of each phase in seconds.

Phases	Phase Name	Duration (S)
1	Preparation	125 ± 95
2	Calot’s triangle dissection	954 ± 538
3	Clipping and cutting	168 ± 152
4	Gallbladder dissection	857 ± 551
5	Gallbladder packaging	98 ± 53
6	Cleaning and coagulation	178 ± 166
7	Gallbladder retraction	83 ± 56

**Table 4:** Evaluation metrics of the model.

	Mean	Standard Deviation
No. of videos	30	30
Accuracy	0.91	0.07
Precision	0.81	0.10
Recall	0.83	0.09

**Table 5:** Results comparison with one Transformer encoder.

	Two-Transformer Encoders Model	One-Transformer Encoder Model
No. of videos	30	30
Accuracy	0.91	0.88

**Table 6:** Results comparison with other related methods.

	Method	Accuracy (%)
Cholec90 Dataset	MTRCNet-CL [25]	89.2
	EndoNet [3]	81.7
	PhaseNet [26]	78.8
	SV-RCNet [27]	85.3
	OHFM [28]	87.3
	Trans-SVNet [29]	90.3
	Ours	91.0

**Table 7:** T-paired analysis for two Transformer models.

	Model 1	Model 2
Accuracy (%)	0.91	0.88
Paried *t*-test	6.12	
Alpha	0.05	
*p*-value	0.02	

## Data Availability

The dataset explored in the research can be found at https://camma.u-strasbg.fr/datasets (accessed on 1 January 2024).
